# Antifeedant activity of luteolin and genistein against the pea aphid, *Acyrthosiphon pisum*

**DOI:** 10.1007/s10340-012-0452-z

**Published:** 2012-06-22

**Authors:** Sylwia Goławska, Iwona Łukasik

**Affiliations:** Department of Biochemistry and Molecular Biology, Siedlce University of Natural Sciences and Humanities, Prusa 12, 08-110 Siedlce, Poland

**Keywords:** Luteolin, Genistein, *Acyrthosiphon pisum*, EPG, Artificial diet, Flavonoid toxicity

## Abstract

Electrical penetration graphs (DC EPG) were used to monitor the feeding behavior of the pea aphid, *Acyrthosiphon pisum* Harris (Hemiptera: Aphididae) exposed to the flavonoids luteolin and genistein in artificial diets. The EPG patterns generated by aphids feeding on plants were used to interpret the patterns generated on the artificial diets. Addition of flavonoids to the diets generally prolonged the period of stylet probing (as indicated by EPG pattern d-C), reduced salivation (as indicated by pattern d-E1) and passive ingestion (as indicated by pattern d-E2), and also delayed the onset of salivation and passive ingestion. At higher concentrations (≥100 μg cm^−3^ for luteolin, ≥1,000 μg cm^−3^ for genistein), the flavonoids completely stopped salivation and passive ingestion. In most events associated with active ingestion (EPG pattern d-G), however, differences in feeding behavior did not statistically differ between the control diet and those with flavonoids; luteolin, and genistein only at 10 μg cm^−3^ prolonged the time until the first d-G pattern was observed. The current findings demonstrate detrimental effects of the isoflavone genistein and the flavone luteolin on the feeding behavior of the pea aphid, *A. pisum*. This can be employed to create plants which are resistant to aphids and other herbivores.

## Introduction

The pea aphid, *Acyrthosiphon pisum* Harris (Hemiptera: Aphididae), is a worldwide pest of economically important legume crops. The pea aphid, which is oligophagous, consists of several biotypes or races living on different legume hosts (pea and broad bean, the red clover, and alfalfa races) (Cuperus et al. 1982; Lane and Walters 1991; Via 1991, 1999; Via and Shaw 1996; and Peccoud et al. 2009a, b). *A. pisum* is a vector of more than 30 viruses, including bean yellow mosaic virus, red clover vein mosaic virus, and pea streak virus (Barnett and Diachun 1986; Jones and Proudlove 1991), all of which reduce the yield of legume crops (Garlinge and Robartson 1998).

Although plant chemicals can be used as biopesticides to control insect pests, aphids are difficult to control because of their unique feeding habits and fast multiplication rates (Majumder et al. 2004). As a consequence, researchers are developing a biotechnological control method in which novel genes from plant sources (including those that encode secondary metabolites) are introduced into plant genomes to enhance the resistance of crop plants to phloem-feeding insects (Rharrabe et al. 2007).

Among the wide array of secondary metabolites synthesized by plants and phenolic compounds, including phenols, saponins, flavonoids, and others, are the most biologically active. These natural products greatly affect plant–insect interactions (Kubo 2006) and can confer resistance against phytophagous insects (Simmonds and Stevenson 2001; Hare 2002a, b; Simmonds 2003; Goławska 2007; Goławska and Łukasik 2009; Goławska et al. 2010). Because phenolic compounds can repulse phytophagous insects or have antifeedant, toxic, and regulatory activity affecting insect physiological processes (Cox 2004; Kubo 2006), they may serve as natural pesticides. They may also promote oxidative stress within aphid tissues (Łukasik 2007; Łukasik et al. 2009, 2011).

Flavonoids occur naturally in plants (Peterson and Dwyer 1998) and are localized in epidermal cells, vacuoles, leaf wax, thalli, and leaf hairs (Cuadra et al. 1997; Gitz et al. 1998; Markham et al. 1998; Olsson et al. 1998; Takahama 2004). Their large variety and their structural diversity and bioactivity make flavonoids especially important among the naturally occurring substances (Harborne 1988). Flavonoids have important roles in plant development and physiology, especially during plant interactions with other organisms (Berhow and Vaughn 1999). Flavonoid glycosides and free aglycones, for example, are involved in pathogenic and symbiotic interactions with microorganisms (Dixon et al. 1994; Spaink 1995) and also affect interactions with insects (Nahrstedt 1989). Most plants contain an array of flavonoids, and evidence suggests that insects are able to discriminate among plants with different flavonoid profiles (Simmonds 2001). Flavonoids can bind to the ecdysone receptor of insects (Oberdorster et al. 2001) and can modulate the feeding behavior of insects and act as feeding deterrents (Morimoto et al. 2000; Knüttel and Fiedler 2001; Van Loon et al. 2002).

Although there has been some research on the effects of flavonoids on insects, there has been very little research on how flavonoids affect insect behavior in general and feeding behavior in particular. In this paper, the effects of flavonoids on pea aphid feeding behavior are examined in detail. Two polyphenolic flavonoids, luteolin, and genistein, were used in in vitro experiments. These flavonoids have been exploited for their beneficial effects on human nutrition (Arai et al. 2000; Erdman et al. 2007; Mink et al. 2007). They also could be useful in a pest management strategy involving transgenic plants that express specific flavonoids. Such genetic engineering is possible which has already been demonstrated. For example, the flavonoid IFS (isoflavone synthase) has been cloned and expressed in Arabidopsis (*Arabidopsis thaliana*), soybean (*Glycine max*), alfalfa (*Medicago sativa*), red clover (*Trifolium pratense*), tobacco (*Nicotiana tabacum*), maize (*Zea mays*), licorice (*Glycyrrhiza echinata*), lettuce (*Lactuca sativa*), and petunia (*Petunia hybrida* *Vilm.*) (Akashi et al. 1999; Jung et al. 2000; Yu et al. 2000, 2003; Kim et al. 2003; Deavours and Dixon 2005; Liu et al. 2007).

Advances in our knowledge about the health benefits of flavonoids in crop and medicinal plants have prompted plant breeders to use traditional and engineering methods to increase the levels of these compounds in crops (Johnson and Felton 2001; Galili et al. 2002). So, we could be creating a world of plants richer in flavonoids. However, the effect of increasing the levels of specific flavonoids in plants on the behavior of insects is unknown. In spite of examinations concerning the activity of flavonoids, their precise mode of insecticidal action is not fully understood. Different flavonoids are thought to have different modes of action on different insects. Researchers have proposed that the mode of insecticidal activity of flavonoids is connected with their effect on insect fitness, insect behavior, physiology, and metabolism. While flavonoids can have insecticidal activity, they could also benefit insect pests and make them more difficult to control with biological control agents such as viral pathogens (Simmonds 2001, 2003).

The current study used the electrical penetration graph (EPG) method to monitor the feeding behavior of pea aphids exposed to the flavonoids luteolin and genistein in an artificial diet. No previous study has examined the effects of these flavonoids on the feeding behavior of the pea aphid. Understanding the activity of these compounds should (1) help researchers overcome the difficulties in using flavonoids to construct transgenic plants that resist insects; (2) clarify the appropriateness of using the candidate genes for a given agronomical purpose (Sauvion et al. 2004). The EPG method detects different waveform patterns related to aphid activities and stylet locations during penetration and feeding (Sauvion et al. 2004; Sauvion and Rahbe 1999). The EPG method was used because it is the only method that can provide continuous information on feeding/probing events.

## Materials and methods

### Aphid culture

The pea aphids (*A. pisum* Harris) used in this study were obtained from a stock culture kept at the Siedlce University of Natural Sciences and Humanities, Poland. The stock culture was maintained on broad beans (*Vicia faba* L. var. Start (Fabaceae)) in plastic pots in an environmental chamber at 21 ± 1 °C and with a L16:D8 photoperiod and 70 % RH. Adult apterous females were used for the experiments. Aphid cohort production was as described earlier (Rahbe and Febway 1993).

### Chemicals and gels

Luteolin were purchased from Sigma-Aldrich (CN. 491703), and genistein was purchased from Fluka (CN. 446720). The effect of flavonoids on pea aphid feeding behavior was investigated in vitro using sucrose–agarose gels. Gels were prepared by incorporating 1.25 % agarose (Sigma A-0169) into a 30 % sucrose solution and then adding one of the flavonoids to obtain concentrations of 0 (control), 10, 100, and 1000 μg cm^−3^. After the mixtures were stirred, they were heated in a water bath (75 °C for 30 min) and then poured into plastic rings (10-mm high and 15-mm diameter) covered with a stretched Parafilm M^®^ membrane. Transparent gels formed after 1–2 min and were offered to aphids for probing.

### EPG recordings

EPGs (Tjallingii 1988) were used to monitor the feeding behavior of the adult aphids that were exposed to flavonoids in an artificial diet. Apterous adults were collected between 6 and 7 a.m. and dorsally tethered on the abdomen with a gold wire (2-cm long, 20 μm in diameter) and water-based conductive silver paint (Demetron, L2027, Darmstadt, Germany). After the aphids were starved for 2 h, they were carefully transferred to the diets. The tethered aphids were individually placed on the surface at the center of each diet, and a second electrode was introduced into the diet. Four aphids were connected to a Giga-4 EPG amplifier and four to the second Giga-4 EPG amplifier (Wageningen Agricultural University, Entomology Department, The Netherlands) coupled to an IBM compatible computer through a DAS 8 SCSI acquisition card (Keithley, USA). U_out_ was 1 Giga Ohm. During EPG recordings, aphids were in a Faraday cage in the laboratory (21 ± 1 °C, L16:D8 photoperiod, and 70 % RH). EPG recordings began between 9 and 10 a.m. on both control and flavonoid diets. EPG recordings were made for 10 aphids on diets without flavonoids (control) and for 10 aphids for each flavonoid concentration (1, 10, 100, or 1000 μg cm^−3^). Aphid feeding behavior was monitored for 2 h.

### EPG analysis

EPGs were acquired and analyzed with STYLET 2.2 software (ref). The main waveform patterns induced by the diets in this study were termed d-np, d-C, d-E1, d-E2, and d-G, previously found and identified in artificial diets (Sauvion and Rahbe 1999, Sauvion et al. 2004; Goławska 2007; Cid and Fereres 2010; Sprawka and Goławska 2010) by analogy to those already defined and described for plants (Tjallingii 1985, 1988, 1990, 1994). In waveform pattern d-np, the aphid’s stylet is outside the diet (analogous to the stylet being outside the plant). Pattern d-C indicates stylet activity in the diet (analogous to the stylet penetrating the epidermis and mesophyll, before salivation and ingestion). Pattern d-E1 indicates salivation into the diet (analogous to the stylet salivating into phloem sieve tubes). Pattern d-E2 indicates passive ingestion of the diet (analogous to the stylet passively ingesting phloem sap). Pattern d-G indicates active ingestion of the diet (analogous to the stylet actively ingesting xylem sap). The 12 behavioral parameters that were measured can be divided into non-sequential parameters (e.g., frequency, total, and average time of patterns) and sequential parameters (e.g., time from the start of the experiment to appearance of the first patterns). The time spent on each EPG parameter was measured in each group and expressed per one insect.

### Statistical analysis

The values of the EPG parameters (the duration of stylet activity in the diet, duration of salivation into the diet, duration of passive and active ingestion from the diet, and the number of probes) were analyzed with the Kruskal–Wallis test in Statistica for Windows version 6.0 (StatSoft Inc. 2003).

## Results

EPG recordings indicated that the addition of the flavonoids luteolin and genistein to the artificial diet clearly affected the feeding behavior of *A. pisum* and that the effect depended on flavonoid concentration (Table 1). Although aphids probed the diet in the controls and in all flavonoid treatments (as indicated by the presence of d-C patterns), aphids did not exhibit salivation and passive ingestion (as indicated by the absence of d-E1 and d-E2 patterns) with diets containing 1,000 μg cm^−3^ of luteolin or genistein. All EPG patterns were observed on diets that contained <100 μg cm^−3^ of luteolin or <1,000 μg cm^−3^ of genistein (Table 1).

**Table 1:** *Acyrthosiphon pisum* feeding behavior on an artificial diet as affected by luteolin and genistein and as indicated by EPG patterns

Flavonoid added	Concentration (μg cm^−3^)	EPG pattern^a^
Control	0	d-C, d-E1, d-E2, d-G
Luteolin	1	d-C, d-E1, d-E2, d-G
	10	d-C, d-E1, d-E2, d-G
	100	d-C, d-E1
	1,000	d-C
Genistein	1	d-C, d-E1, d-E2, d-G
	10	d-C, d-E1, d-E2, d-G
	100	d-C, d-E1, d-E2, d-G
	1,000	d-C, d-G

^a^ *d-C* indicates stylet penetration of the diet and is analogous to stylet penetration of the plant tissues; *d-E1* indicates salivation into the diet and is analogous to the excretion of saliva into the phloem; *d-E2* indicates passive ingestion of the diet and is analogous to the ingestion of phloem sap; *d-G* indicates active ingestion of the diet and is analogous to the ingestion of xylem sap

Although the d-C pattern was exhibited on all diets (Table 1), and although the number of penetrations was unaffected by the treatments (Table 2), the timing of the first probe was prolonged by genistein at 1,000 μg cm^−3^ and tended to be prolonged, but without statistical significance, by the higher concentrations of luteolin (Table 2). The average time of probing also tended to be higher with addition of the flavonoids but the effect was not statistically significant (Table 2).

**Table 2:** *Acyrthosiphon pisum* stylet activity (d-C pattern) on an artificial diet as affected by luteolin and genistein

Flavonoid added	Concentration (μg cm^−3^)	Number of penetrations/2 h	Time the first probing (min)	Average time of probing (min)
Control		3.20 ± 1.47a	7.23 ± 5.81b	8.88 ± 5.86a
Luteolin	1	4.00 ± 0.45a	19.97 ± 1.21ab	18.86 ± 2.41a
	10	3.50 ± 0.65a	17.92 ± 7.51ab	20.33 ± 7.11a
	100	4.40 ± 1.12a	33.31 ± 8.62ab	27.36 ± 5.72a
	1,000	4.60 ± 0.96a	21.28 ± 7.13ab	33.29 ± 5.52a
Genistein	1	2.80 ± 0.01a	2.95 ± 0.36b	5.49 ± 0.36a
	10	2.90 ± 0.35a	6.92 ± 3.62ab	9.74 ± 2.93a
	100	4.00 ± 0.93a	27.08 ± 6.87ab	18.70 ± 4.97a
	1,000	3.40 ± 0.48a	52.13 ± 4.23a	36.44 ± 5.94a

Values were derived from 2-h EPG recordings and are means ± SE; *n* = 10. Means in columns followed by different letters are different at *P* < 0.05 (Kruskal–Wallis test)

The higher concentrations of luteolin and genistein (100 and 1,000 μg cm^−3^) reduced or completely inhibited aphid salivation (pattern d-E1) and passive ingestion (pattern d-E2) (Table 3). Both flavonoids at 1,000 μg cm^−3^ reduced the total time that pea aphids salivated into the diets (Table 3). A similar tendency was observed for passive ingestion from diets for both flavonoids at 100 and 1,000 μg cm^−3^ (Table 3). Genistein at 100 μg cm^−3^ reduced the duration of passive ingestion up to 60 times, and no passive ingestion occurred with luteolin at 100 μg cm^−3^ or with luteolin or genistein at 1,000 μg cm^−3^. This phase of aphid feeding was generally reduced at the lower concentrations of both flavonoids and was completely stopped by the higher concentrations (Table 3). In the time until first d-E1 and time the first d-E1, the statistical differences were observed for both flavonoids at 1,000 μg cm^−3^. In the time until first d-E2, and time the first d-E2 patterns, the statistical differences were observed for luteolin at 100 and 1,000 μg cm^−3^ and for genistein at 1,000 μg cm^−3^ (Table 3).

**Table 3:** *Acyrthosiphon pisum* salivation (pattern d-E1) and passive ingestion (pattern d-E2) on an artificial diet as affected by luteolin and genistein

Aphid activity (in min)	Control	Concentration of flavonoid (μg cm^−3^)							
		1		10		100		1,000	
		Luteolin	Genistein	Luteolin	Genistein	Luteolin	Genistein	Luteolin	Genistein
Time until first d-E1 pattern	16.74 ± 7.36a	20.38 ± 1.23a	12.99 ± 0.23a	18.18 ± 7.56a	15.65 ± 7.77a	46.29 ± 11.18a	17.47 ± 7.49ab	0.00 ± 0.00b	0.00 ± 0.00b
Time the first d-E1 pattern	13.53 ± 6.46a	6.00 ± 0.08a	8.57 ± 0.75a	18.45 ± 5.39a	6.77 ± 1.18a	5.44 ± 2.17a	2.92 ± 1.45ab	0.00 ± 0.00b	0.00 ± 0.00b
Total time of d-E1 pattern	18.74 ± 6.51a	16.29 ± 1.38a	17.19 ± 0.82a	31.20 ± 6.69a	11.41 ± 2.94a	13.57 ± 5.75ab	9.94 ± 4.22ab	0.00 ± 0.00b	0.00 ± 0.00b
Time until first d-E2 pattern	30.27 ± 7.89a	30.88 ± 1.15a	17.16 ± 0.52abc	39.39 ± 7.49a	26.79 ± 7.61ab	0.00 ± 0.00c	26.47 ± 9.94ac	0.00 ± 0.00c	0.00 ± 0.00c
Time the first d-E2 pattern	24.26 ± 8.35a	13.29 ± 2.27a	39.86 ± 1.55a	8.51 ± 2.10a	11.69 ± 1.86a	0.00 ± 0.00bc	1.18 ± 0.42abc	0.00 ± 0.00bc	0.00 ± 0.00c
Total time of d-E2 pattern	60.17 ± 8.36a	19.88 ± 2.67ab	54.00 ± 1.55ab	16.40 ± 3.70ab	16.39 ± 1.89ab	0.00 ± 0.00c	1.26 ± 0.47bc	0.00 ± 0.00c	0.00 ± 0.00c

Values were derived from 2-h EPG recordings and are means ± SE; n = 10. Means in rows followed by different letters are different at *P* < 0.05 (Kruskal–Wallis test)

As indicated by the d-G pattern, the flavonoids tended to delay, prolong, or inhibit active ingestion, but the differences were significant in only one case: luteolin and genistein at 10 μg cm^−3^ prolonged the duration until the first d-G pattern was detected (Table 4).

**Table 4:** *Acyrthosiphon pisum* active ingestion (pattern d-G) on an artificial diet as affected by luteolin and genistein

Aphid activity (in min)	Control	Concentration of flavonoid (μg cm^−3^)							
		1		10		100		1,000	
		Luteolin	Genistein	Luteolin	Genistein	Luteolin	Genistein	Luteolin	Genistein
Time until first d-G pattern	6.85 ± 5.73 cd	57.59 ± 8.86abc	12.07 ± 0.05bcd	70.44 ± 8.62a	54.01 ± 6.11ab	0.00 ± 0.00d	62.34 ± 8.39abc	0.00 ± 0.00d	51.14 ± 12.54abcd
Time the first d-G pattern	16.35 ± 9.94bcd	28.09 ± 4.56abc	15.06 ± 0.05 cd	25.98 ± 5.09abc	37.32 ± 5.56abc	0.00 ± 0.00d	43.00 ± 11.30ac	0.00 ± 0.00d	8.38 ± 2.91 cd
Total time of d-G pattern	22.87 ± 10.28ab	32.70 ± 4.56ab	26.72 ± 0.03ab	34.75 ± 4.74a	61.08 ± 6.99a	0.00 ± 0.00b	56.63 ± 8.94a	0.00 ± 0.00b	19.50 ± 6.15ab

Values were derived from 2-h EPG recordings and are means ± SE; *n* = 10. Values in rows followed by different letters are different at *P* < 0.05 (Kruskal–Wallis test)

## Discussion

Our EPG recordings demonstrated that pea aphid feeding behaviors on sucrose-agarose gels were clearly affected by the flavonoids luteolin and genistein. The EPG results indicated that the flavonoids reduced aphid ingestion. Luteolin at >100 μg cm^−3^ and genistein at 1,000 μg cm^−3^ blocked passive ingestion of the diet (i.e., no E2 pattern was detected). The current study represents an initial step in this area of research because there are a few data directly concerned with the effect of flavonoids on the feeding behavior of insects. We used different concentrations of flavonoids because LC_50_ values for these molecules have not been determined for *A. pisum*. Moreover, plants generally contain a great diversity of flavonoids, and flavonoid profiles, and levels often differ among families, genera, and species (Harborne and Turner 1984). Although flavonoids can clearly be involved in different stages of insect–plant interactions, it is still difficult to predict how flavonoids in plants might influence insect feeding (Simmonds 2001).

Although the effects of flavonoids on aphid development and fecundity were not studied in our experiment, negative effects of flavonoids on herbivore performance (e.g., reduced growth, pupal mass, and fecundity, and increased mortality) were previously demonstrated (Ruuhola et al. 2001; Alonso et al. 2002; Hare 2002a, b). In a review of chemicals that impart natural resistance to insect attack in wood, Rao (1982) reported that flavonoids including isoflavonoids were important. Flavonoid aglycones reduced the growth rate and prolonged the duration of the first instar larvae of *Epirrita autumnata* (Lahtinen et al. 2004). Contents of both total flavonoid and individual flavonoid compounds have been also shown to reduce the larval performance of certain mid-to-late and late sawfly species (Lahtinen et al. 2006). Some flavonoids can either stimulate insect feeding (Bernays et al. 1991) or act as feeding deterrents (Morimoto et al. 2000). They can act as endocrine disruptors in mammalian systems, having high binding affinities for estrogen receptors, and flavonoids have recently been shown to bind to the ecdysone receptor of insects (Oberdorster et al. 2001). Boué and Raina (2003) found that of five flavonoids tested at 50 μg, only the isoflavone genistein significantly reduced the number of progeny produced by the Formosan subterranean termite within 30 days after treatment. In endocrine disruptor studies, isoflavones are more active than flavones, particularly in their ability to bind to the estrogen receptor in mammalian systems, and have been shown to disrupt animal reproduction (Shutt 1976; Setchell et al. 1987).

In our study, the flavone luteolin was more active than the isoflavone genistein in disrupting pea aphid feeding behavior. Passive ingestion (as indicated by the E2 pattern) was completely blocked by luteolin at 100 μg cm^−3^ and by genistein at 1,000 μg cm^−3^. In insects, flavonoids interfere with molting, reproduction, feeding, and behavior (Beninger and Abou-Zaid 1997; Musayimana et al. 2001; Simmonds 2001). Insecticidal activity of flavonoids has been documented against the western corn rootworm (Mullin et al. 1992), the corn earworm (Widstrom and Snook 2001), and the common cutworm (Morimoto et al. 2000). Reyes-Chilpa et al. (1995) determined that two flavonoids, castillen D and E, showed concentration-dependent feeding deterrence against *Cryptotermes brevis*. Blaney and Simmonds (1983) found that rutin at concentrations >10^−3^ M deterred the final stadium larvae of *Heliothis zea* and *Heliocoverpa armigera* from feeding. Simmonds (2003) reviewed the antifeedant activity of flavonoids against insects.

The results presented here suggest that the negative effects of flavonoids on the performance of aphids and perhaps of other insect herbivores could be a consequence of shortening or suppressing the feeding process. Furthermore, these results support the hypothesis that the mode of insecticidal activity of flavonoids is associated with their influence on insect feeding behavior (Simmonds 2001). Frazier and Chyb (1995) suggested that insect feeding can be inhibited at three levels: the preingestional level (an immediate effect associated with host finding and host selection processes involving gustatory receptors); the ingestional level (related to food transport and the production, release, and activity of salivary enzymes); and the postingestional level (long-term effects involving various aspects of digestion and absorption of food). Because aphid probing behavior cannot be observed directly, EPG recordings are used to measure the effect of various factors on preingestional and ingestional processes. In this study, the flavonoids luteolin and genistein deterred aphid probing and feeding. On diets containing flavonoids, the time spent penetrating the diet (the d-C pattern) was long relative to the total penetration time, the time until the first salivation and passive ingestion from diets was prolonged, and the duration of passive ingestion was short. These indicate that the flavonoids acted as antifeedants.

Detailed understanding of how flavonoids modulate behavior, especially feeding behavior, remains unknown (Simmonds 2003). Flavonoids and isoflavonoids, which are synthesized by plants via the phenylpropanoid pathway (the key enzyme is phenylalanine ammonia-lyase = PAL), contribute to plant defense against stressors (Dakora and Phillips 1996) such as pathogens, herbivores, or abiotic factors. Hagerman and Butler (1991) showed that plant wounding also induces these compounds. Previous research indicated that individual flavonoids in artificial diets could be detrimental to insect growth by virtue of their prooxidant properties (Ahmad and Pardini 1990). Although Johnson and Felton (2001) indicated that proportion of individual flavonoids are important in determining activity, Simmonds and Stevenson (2001) and Yu et al. (2003) showed that isoflavonoids irrespective of composition can have negative effects on herbivores. Our study indicates that the flavonoids luteolin and genistein have detrimental effects on aphid feeding. The results confirm the reports by Cipollini et al. (2008) and O’Nill et al. (2010), who suggested that luteolin and genistein deterred the feeding of the generalist herbivores *Spodoptera exigua*, *Popillia japonica*, *Aphis glycines*, and *Vanessa cardui*.

In summary, this work documents detrimental effects of the isoflavone genistein and the flavone luteolin on the feeding behavior of the pea aphid, *A. pisum*. Although these chemicals may have potential for *A. pisum* control, further investigation on their precise modes of activity and biological effects are needed if we are to use these compounds for creating transgenic plants that are resistant to aphids and other herbivores.
